# Reference genes for quantitative reverse transcription-polymerase chain reaction expression studies in wild and cultivated peanut

**DOI:** 10.1186/1756-0500-4-339

**Published:** 2011-09-09

**Authors:** Carolina V Morgante, Patricia M Guimarães, Andressa CQ Martins, Ana CG Araújo, Soraya CM Leal-Bertioli, David J Bertioli, Ana CM Brasileiro

**Affiliations:** 1 EMBRAPA Recursos Genéticos e Biotecnologia. Parque Estação Biológica, CP 02372. Final W5 Norte, Brasília, DF - Brazil; 2 EMBRAPA Semiárido, CP 23, Petrolina, PE - Brazil; 3 Universidade de Brasília, Campus I, Brasília, DF - Brazil; 4 Universidade Católica de Brasília, Campus II, 916 Norte, Brasília, DF - Brazil

## Abstract

**Background:**

Wild peanut species (*Arachis *spp.) are a rich source of new alleles for peanut improvement. Plant transcriptome analysis under specific experimental conditions helps the understanding of cellular processes related, for instance, to development, stress response, and crop yield. The validation of these studies has been generally accomplished by quantitative reverse transcription-polymerase chain reaction (qRT-PCR) which requires normalization of mRNA levels among samples. This can be achieved by comparing the expression ratio between a gene of interest and a reference gene which is constitutively expressed. Nowadays there is a lack of appropriate reference genes for both wild and cultivated *Arachis*. The identification of such genes would allow a consistent analysis of qRT-PCR data and speed up candidate gene validation in peanut.

**Results:**

A set of ten reference genes were analyzed in four *Arachis *species (*A. magna*; *A. duranensis*; *A. stenosperma *and *A. hypogaea*) subjected to biotic (root-knot nematode and leaf spot fungus) and abiotic (drought) stresses, in two distinct plant organs (roots and leaves). By the use of three programs (GeNorm, NormFinder and BestKeeper) and taking into account the entire dataset, five of these ten genes, *ACT1 *(actin depolymerizing factor-like protein), *UBI1 *(polyubiquitin), *GAPDH *(glyceraldehyde-3-phosphate dehydrogenase), *60S *(60S ribosomal protein L10) and *UBI2 *(ubiquitin/ribosomal protein S27a) emerged as top reference genes, with their stability varying in eight subsets. The former three genes were the most stable across all species, organs and treatments studied.

**Conclusions:**

This first in-depth study of reference genes validation in wild *Arachis *species will allow the use of specific combinations of secure and stable reference genes in qRT-PCR assays. The use of these appropriate references characterized here should improve the accuracy and reliability of gene expression analysis in both wild and cultivated Arachis and contribute for the better understanding of gene expression in, for instance, stress tolerance/resistance mechanisms in plants.

## Background

Cultivated peanut (*Arachis hypogaea*) is one of the most widely grown grain legumes in the world, thanks to its high protein and unsaturated oil contents [1]. It is grown extensively in Asia, Africa, United States and Latin America, but is subject to attacks from various pests and diseases, necessitating substantial pesticide use. By contrast, wild *Arachis *species, which are exclusively South American in origin, are a rich source of new alleles for peanut improvement, with sufficient polymorphism for their genetic characterization [2-4]. Basic resources for gene discovery, interpretation of genomic sequences and marker development have been developed for a number of wild *Arachis *species [5-7], and constitute important tools for the analysis of the complexities of gene expression patterns and functions of transcripts in *Arachis*. Additionally, recent research has identified a number of stress responsive genes from wild and cultivated *Arachis*. These genes, generated by several research groups, are candidate disease resistance and drought tolerance genes and need further analysis to be validated [2,7-12]. The use of a common set of standards would help in the comparison of research results generated in different labs.

Quantitative reverse transcription-polymerase chain reaction (qRT-PCR) is currently the most sensitive technique for quantification of low abundance transcripts, and at the same time is suitable for abundant transcripts. For these reasons, and because of relative ease of use, qRT-PCR has become widely preferred to classic transcriptome analysis tools, such as Northern blotting, semi-quantitative RT-PCR, micro and macroarrays, RNase protection analysis, and *in situ *hybridization [13,14]. qRT-PCR technology can be either used to quantify with extremely high sensitivity the input copy number of a particular transcript (absolute quantification) or to measure the change in expression of a target gene relative to a reference gene (relative quantification). By far, the latter is the analytic method of choice for the majority of gene expression studies as it is usually unnecessary to know the absolute transcript copy number. The method continues to be improved, with recent developments enabling qRT-PCR reactions to be performed at lower reagents cost, less hands-on time and with higher throughput than previously possible [15,16].

Nevertheless, in spite of these advantages there are a number of variables that strongly interfere with the accuracy and reliability of qRT-PCR. These include initial sample amount, RNA recovery, RNA integrity, efficiency of cDNA synthesis, and differences in the overall transcriptional activity of the tissues or cells analyzed [17,18]. The effect of all of these variables can be largely corrected for by the normalization of mRNA levels among samples. Different approaches have been proposed for the normalization of expression level measurements, but it is generally done by using an internal 'reference gene', under the assumption that this has a constant level of expression in the chosen tissue, is not affected by the treatment, and has no inter-individual variability [14,17-19].

Reference control genes have been identified for several plant species [15,16,20-26]. However, a number of studies reported that some of the most common internal control genes such as β-actin, glyceraldehyde-3-phosphate dehydrogenase (*GAPDH*), 18S or 26S ribosomal RNA and α-tubulin were expressed irregularly and unsteadily in some experiments, questioning the concept of an ideal, universal internal control gene [19,27,28]. In fact, it is now a consensus that it is almost impossible to obtain only one invariable gene, and that multiple internal control genes must be evaluated and utilized to quantify gene expression, in order to improve the accuracy of a qRT-PCR analysis and interpretation [15,22,29].

Recently, reference genes for qRT-PCR have been analyzed on a set of five tissues (full pod; mature seed; leaf; gynophores; and root) of cultivated peanut (*A. hypogaea*) showing some intra- and inter-tissue variation in gene stability [30]. Ten generally used housekeeping primers for reference genes were designed for peanut and analyzed by GeNorm and NormFinder programs. Alcohol dehydrogenase (*ADH3*) showed to be the most stably expressed gene across samples, followed by 60S ribosomal protein L7 (*60S*) and yellow leaf specific 8 (*YLS8*) [30]. However, to date, no endogenous control genes have been identified for other *Arachis *species, including the wild relatives which constitute a source of resistances to biotic and environmental constraints. In the present work, a simplified qRT-PCR protocol based on SYBR reagent was used for the identification of genes with minimal expression variation in four *Arachis *species (*A. magna*; *A. duranensis*; *A. stenosperma *and *A. hypogaea*) subjected to biotic (*Meloidogyne arenaria*, *Cercosporidium personatum*) and abiotic (drought) stresses in roots and leaves. For that, we used our ESTs databank of wild *Arachis *[7] to survey for potential internal control genes and three distinct programs (GeNorm, NormFinder, and BestKeeper) for their evaluation. Our data show that the combined use of these new internal control genes for normalization of target gene expression in qRT-PCR improves the accuracy and reliability of the analysis of gene expression in different species of the genus *Arachis *under different stresses.

## Methods

### Plant materials and bioassays

*Arachis stenosperma *(accession V10309), *A. magna *(accession KG30097), *A. duranensis *(accession K7988), and *A. hypogaea *(cultivar IAC- Tatu - ST) seeds were obtained from the Active Germplasm Bank at Embrapa Genetic Resources and Biotechnology-Cenargen (Brasília, Brazil). Plants were kept in open plan greenhouse and treatments were imposed at the 30-leaf stage. For the leaf spot fungi (*C. personatum*) bioassays, ten plants of each, the resistant (*A. stenosperma*) and susceptible genotypes (*A. duranensis *and *A. hypogaea*), were inoculated with a of 50,000 spores/mL suspension diluted in Tween 20, as previously described [31]. Leaves and roots were collected from inoculated and non-inoculated plants 72 hours after inoculation (HAI). For nematode challenge, ten plants of nematode-resistant *A. stenosperma *and the susceptible cultivated *A. hypogaea *were inoculated with 10,000 root-knot nematode *M. arenaria *race 1 juveniles (J2), as previously described [32,33]. Roots from challenged and non-challenged plants were collected nine days after inoculation (DAI). For abiotic stress assays, ten plants of drought tolerant species *A. magna *and *A. duranensis *were subjected to gradual water deficit in soil whilst control plants remained at 90% field capacity. Individual Normalized Transpiration Ratio (NTR) was calculated essentially as described by Sinclair and Ludlow [34] and leaves and roots were collected when plants reached an average NTR of 0.5.

### RNA purification and cDNA synthesis

Collected leaves and roots from stressed and control plants were immediately frozen in liquid nitrogen and stored at -80°C. Total RNA was extracted from 250 mg of plant material using a modified lithium chloride protocol [35] with an additional RNA precipitation step (3M sodium acetate and ethanol 96%), followed by purification on Invisorb Spin Plant RNA Mini columns (Invitek, Berlin, Germany) to eliminate impurities. RNA integrity was checked by gel electrophoresis. Total RNA was quantified at 260 nm using the NanoDrop^® ^ND-1000 spectrophotometer (Thermo Scientific, Waltham, USA) and its purity confirmed as a 260/280 nm ratio above 1.8. Each sample contained 2 μg of total RNA and comprised a pool of equal RNA quantities of all individuals collected at the same point.

Thus, a total of 24 samples was examined in this study, representing the three stress conditions tested: (i) Fungus bioassay: three species (*A. stenosperma*, *A. duranensis *and *A. hypogaea*); two plant organs (roots and leaves) and two treatments (inoculated and non-inoculated); total of 12 samples; (ii) Nematode bioassay: two species (*A. stenosperma *and *A. hypogaea*); one plant organ (roots) and two treatments (inoculated and non-inoculated); total of four samples; and (iii) Drought stress: two species (*A. duranensis *and *A. magna*); two plant organs (roots and leaves) and two treatments (stressed and non-stressed); total of eight samples.

After sampling, DNAse treatment and cDNA synthesis were carried out in subsequent steps, in the same tube. Genomic DNA contaminants were removed from total RNA by treatment with DNase (TURBO DNA-free™, Ambion, USA), according to the manufacturer's instruction, followed by first strand cDNA synthesis performed at 42°C for 60 min on a Master Cycler thermocycler (Eppendorf AG, Hamburg, Germany) using SuperScriptTM II RT and Anchored Oligo(dT)_20 _primer (Invitrogen, Carlsbad, CA, USA), according to the manufacturer's instruction. Both enzymes (DNase and Reverse Transcriptase) were heat inactivated in the tube and the resulted cDNA was directly used in qRT-PCR assays.

DNA contamination in cDNA samples was checked by RT-PCR using a pair of conserved primers flanking an intron region in *Arachis *(Leg066Fwd-5'AGCTCCACCTCTTTCCGACAGA3' and Leg066Rev-5' AGTTTCTACAGCACGTATCCTTTCC3'), as previously described [5,36], which allows the distinction between PCR products amplified from genomic DNA and cDNA templates.

### PCR primer design

Ten *Arachis *candidate genes were selected based on their previous description as good plant internal control genes for qRT-PCR analysis in a number of species [21,22,24,25,28]. Nine of these selected genes were retrieved from our wild *Arachis *EST libraries (*A. magna *and *A. stenosperma*) and from *A. hypogaea *database available at GenBank (Table 1), whilst *UBI2 *was included as it was previously used as a reference gene in *A. hypogaea *gene expression qRT-PCR analysis [10]. Amplification primers for qRT-PCR were designed with Primer3Plus software [37], using the following parameters: amplicon length between 150 and 200 bp; size between 19 and 22 bp; melting temperature (Tm) between 59 and 61°C; GC content between 40 and 55%. Amplicon length of selected primers was checked by RT-PCR using as template an equimolar pool of all 24 samples, according to the parameters described above.

**Table 1:** Genes and primers used for qRT-PCR analysis

Gene Abbreviation	*Arachis* species	GenBank ID	Gene description	Primer sequence Forward/ Reverse	Amplicon size (bp)	PCR efficiency (%)	Regression coefficient R^2^
*60S*	*A. stenosperma*	EH042095.1	60S ribosomal protein L10	TGGAGTGAGAGGTGCATTTG/ TCTTTTGACGACCAGGGAAC	155	99.872	0.994

*ACT1*	*A. magna*	Not available	Actin depolymerizing factor-like protein	TGGTCTCGGTTTCCTGAGTT/ AATACCACTCCAAAGCAAACG	114	98.330	1.000

*ACT2*	*A. hypogaea*	GO326795.1	Actin	GAGCTGAAAGATTCCGATGC/ GCAATGCCTGGGAACATAGT	178	108.360	0.994

*EFA*	*A. stenosperma*	EH046450.1	Chloroplast elongation factor tub	CGATGTCACTGGCAAGGTTA/ TAGCGAACCTCATTCCCTGT	137	101.936	1.000

*GAPDH*	*A. magna*	Not available	Glyceraldehyde-3- phosphate dehydrogenase	CAACAACGGAGACATCAACG/ ATCACTGCCACCCAGAAAAC	190	91.802	0.958

*MAN*	*A. stenosperma*	EH048114.1	Mannose/glucose-binding lectin	ATTAAATCCGCTGCAACCAC/ AATCCAACCATACCCCATTC	185	92.192	1.000

*PRO*	*A. stenosperma*	EH047960.1	Proline-rich protein precursor	GCACCCAATTGAAAAACCAC/ GAGGGTACTTGCCATGAGGA	185	90.180	1.000

*TUB*	*A. stenosperma*	EH047237.1	Beta-tubulin	AGTCAGGTGCGGGTAACAAC/ CCAGTACCACCTCCCAAAGA	151	97.668	1.000

*UBI1*	*A. stenosperma*	EH047293.1	Polyubiquitin	TCTTGTCCTCCGTCTTAGGG/ AGCAAGGGTCCTTCCATCTT	196	99.997	0.999

UBI2*	*A. hypogaea*	HO115753.1	Ubiquitin/ribosomal protein S27a	AAGCCGAAGAAGATCAAGCAC/ GGTTAGCCATGAAGGTTCCAG	145	99.218	0.999

* Primer pair previously described [10].

### Real-Time PCR conditions

Real-time reactions used Platinum^® ^SYBR^® ^Green qPCR Super Mix-UDG w/ROX kit (Invitrogen, Carlsbad, CA, USA) as follows: 2 μL of cDNA diluted 10 times, 5 μL of the mix and 0.2 μM of each primer, in a final volume of 10 μL. Reactions were carried out using three independent technical replicates for each sample and, to certify the absence of genomic DNA in RNA samples, NAC (No Amplification Control) was carried out using total RNA as reaction template. The StepOne system (Applied Biosystems) was used and PCR cycling consisted of four steps: 50°C for 2 min, 95°C for 10 min, 40 cycles of 95°C for 15 s and 60°C for 1 min, and a final dissociation curve step of 95°C for 15 s, 60°C for 60 s, and 95°C for 15 s. The amplification efficiencies and correlation coefficients R^2 ^values were calculated by standard curve method using as a template an equimolar pool of all samples. Two independent biological replicates for each of the 24 samples were used for real-time PCR analysis, with each replicate representing a pool of five plants.

### Result analysis

Expression levels were assessed based on the number of amplification cycles needed to reach a fixed threshold (Cq) in the exponential phase of PCR. Cq values were converted to relative quantities using the delta-Cq method. The sample with the lowest Cq was used as calibrator and amplification efficiency was incorporated in the analysis. Stability of reference gene expression was analyzed with GeNorm v3.4 [29], NormFinder [17] and BestKeeper [38] tools. GeNorm calculates an average expression stability value (M) based on the geometric averaging of multiple candidate genes and mean pairwise variation existing between all pairs of candidate genes. Genes with the lowest M values have the most stable expression. In addition, GeNorm software also calculates the pairwise variation (Vn/n +1) to indicate the optimal number of reference genes required for normalization. NormFinder software is based on a variance estimation approach and also calculates an expression stability value (M) for each gene analyzed. It enables estimation of the overall variation of the reference normalization genes and the variation between subgroups of the sample set, taking into account intra and intergroup variations for normalization factor (NF) calculations. BestKeeper program indicates the best reference gene by the pairwise correlation analysis of all pairs of candidate genes and calculates the geometric mean of the best suited ones. Reference genes with standard deviation (SD) values greater than 1 are considered by BestKeeper as inconsistent and should be excluded.

For reference gene validation, statistical analyses between Cq values were performed with R software 2.12.0 http://www.r-project.org and REST software was used for relative expression profile and the linear regression analyses [39].

## Results and discussion

### RNA quality and cDNA synthesis

A set of 24 pooled samples including two different tissues (root and leaves) of four *Arachis *species submitted to three different stresses was used to analyze the expression stability of ten candidate genes for normalization of qRT-PCR. Total RNA extracted from wild *Arachis *species was highly viscous, suggesting contamination with polysaccharides and/or other polymers. Therefore, the use of a modified LiCl protocol [35] and an additional column purification step were required to produce good yields of intact and good quality RNA.

Performing the DNase treatment and cDNA synthesis in the same tube produced a higher yield of cDNA of improved quality for qRT-PCR reactions and reduced the loss of RNA or cDNA during the precipitation and washing steps, being a viable alternative for materials with limited amounts of initial RNA. This procedure also generated cDNA samples without genomic DNA contamination.

### Analysis of Cq variability and PCR efficiency

**Figure 1:**
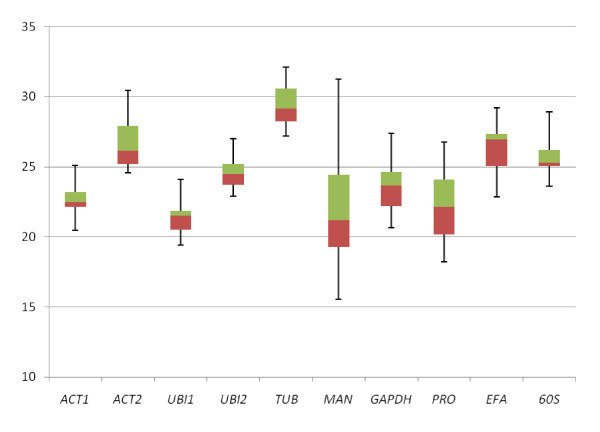
**Cq values distribution of candidate reference genes**. Cq values distribution of the ten candidate reference genes. Values are given as qRT-PCR quantification cycle (Cq). The boxes represent the upper (green) and lower (red) quartiles with medians.

The expression level of the genes tested differed and, in qRT-PCR, they reached fixed thresholds at medians Cq values ranging from 21 to 29, with most lying between 22 and 26 (Figure 1). *UBI1 *and *MAN *were the most expressed genes and *TUB *the least. Standard curves were generated for each pair of primers using an equimolar pool of all cDNA samples in ten-fold serial dilutions. No amplification was detected in the absence of template. The amplification efficiency of the reactions was estimated based on the calculated slopes of the curves, which ranged from 90.2 to 108.4%, with the correlation coefficients R^2 ^varying from 0.958 to 1.000 (Table 1), both within the range expected for a qPCR reaction [40]. For all genes analyzed, single peaked melting curves were generated (Additional file 1), indicating the presence of a specific amplicon and the absence of primer-dimer formation. The values of primer pair efficiencies were used in subsequent qRT-PCR analysis.

### Expression stability of candidate genes

In order to evaluate the stability of the selected candidate reference genes, the level of transcript accumulation of the samples was verified with respect to biotic and abiotic stress, roots and leaves and four *Arachis *species (*A. duranensis, A. stenosperma, A. magna*, and *A. hypogaea*). The data was analyzed considering all samples together and in separate groups (organs, type of stress and species). The expression stability of the ten candidate genes was evaluated by three different softwares: GeNorm, NormFinder, and BestKeeper enabling a more comprehensive analysis of the gene expression data.

**Figure 2:**
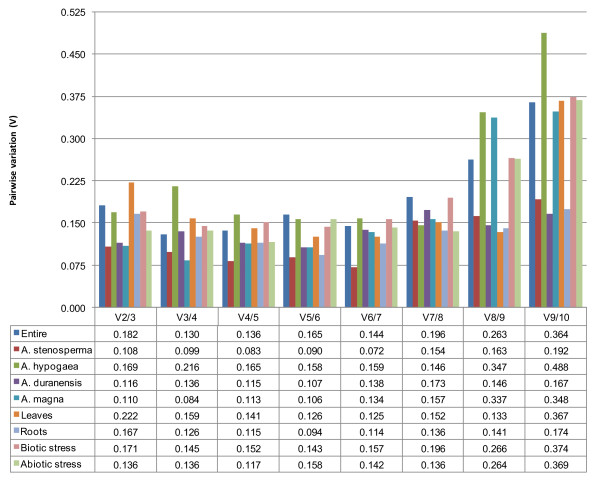
**Pairwise variation of candidate genes as predicted by GeNorm**. Pairwise variation of the ten candidate genes as predicted by GeNorm. The pairwise variation (Vn/Vn+1) was calculated between the normalization factors NFn and NFn+1, with a recommended cutoff threshold of 0.150.

Taking into account the entire dataset, for all species, organs and stresses, *ACT1 *and *UBI1 *(M = 0.553) were the most stable genes by GeNorm analysis (Table 2). Among the selected genes, only *MAN *did not reach high expression stability (M = 1.865), with M value above the default limit of M = 1.5 [29] (Additional files 2 and 3). The pairwise variation V3/4 value (0.130) for the entire dataset was smaller than the recommended cutoff value of 0.150 (Figure 2), below which the inclusion of an additional reference gene is not required [29]. It indicates that the top three ranked genes (*ACT1*, *UBI1*, and *UBI2*) in GeNorm software should be used for qRT-PCR normalization (Figure 2; Additional files 2 and 3). BestKeeper program also indicated *ACT1 *(SD = 0.871) as the gene with the most stable expression (Table 2). On the other hand, six out of the ten genes analyzed (*EFA*, *TUB*, *GAPDH*, *ACT2*, *MAN*, and *PRO*) showed SD values higher than 1, which is an indication that these genes have an unstable expression, according to BestKeeper software (Additional file 3) [38]. NormFinder software highlighted *GAPDH *as the best reference gene (M = 0.056), and ranked *UBI1 *(M = 0.090) and *ACT1 *(M = 0.118) in the second and third positions, respectively (Table 2; Additional file 3).

**Table 2:** Optimal reference genes for quantification of the entire dataset and individual (species, organs or stress) subsets

Program	Entire	Subsets							
		
		Species				Organ		Stress	
		
		*A. stenosperma*	*A. hypogaea*	*A. duranensis*	*A. magna*	Leaves	Roots	Biotic stress	Abiotic stress
GeNorm (M)	*ACT1*/*UBI1 *(0.553)	*ACT1*/*60S *(0.269)	*ACT1*/*UBI1 *(0.535)	*ACT1*/*UBI2 *(0.350)	*UBI2*/*60S *(0.242)	*ACT1*/*UBI1 *(0.483)	*UBI2*/*60S *(0.492)	*ACT1*/*60S *(0.549)	*UBI2*/*60S *(0.376)

NormFinder (M)	*GAPDH *(0.056)	*ACT1* (0.062)	*60S* (0.045)	*60S* (0.057)	*ACT2*/*PRO *(0.013)	*ACT1 *(0.090)	*GAPDH *(0.063)	*GAPDH *(0.076)	*GAPDH* (0.091)

BestKeeper (SD)	*ACT1 *(0.871)	*60S* (0.284)	*UBI2* (0.661)	*EFA* (0.677)	*UBI1 *(0.623)	*UBI2 *(0.603)	*ACT1 *(0.524)	*ACT1* (0.945)	*UBI1* (0.464)

Numbers in parentheses represent expression stability value (M) calculated by GeNorm and NormFinder programs and standard deviation (SD) calculated by BestKeeper program.

The only previous work that assessed reference genes for qRT-PCR in *Arachis *[30] analyzed exclusively the cultivated *A. hypogaea *species in five tissues, including roots and leaves. Overall, taking into account all tissues and treatments, this study concluded that *ADH3*, *60S *and *YLS8 *were the most appropriate reference genes in expression analysis involving seed development. However, in contrast with our analysis, the previously mentioned study [30] considered ubiquitin as an unstable gene that should be avoided in expression studies. A possible reason for this apparently contradictory result is the difference on set composition between the two studies which included different species, treatments and tissues. Our study focused on other species and treatments, and therefore is complementary to Brand and Hovav [30]. This reinforces the need of detailed reference gene analysis for specific plant species, experimental conditions and tissues and also corroborates the general belief that is essential to apply different reference genes for a more accurate and reliable normalization [15,22,29].

### Species subsets

Considering each species separately (species subsets), GeNorm and NormFinder also pointed out *ACT1 *(M = 0.269 and 0.062, respectively) as the best reference gene for *A. stenosperma *(Table 2). All the ten genes had an M value below the GeNorm 1.5 threshold of for this species (Additional files 2 and 3). The pairwise variation V2/3 value (0.108) indicated the use of the two top ranked genes (*ACT1 *and *60S*) for normalization (Figure 2; Table 2). BestKeeper ranked *ACT1 *in the second position (SD = 0.343), and *60S *in the first position (SD = 0.284). This result is quite similar to that obtained by GeNorm, which ranked *ACT1 *and *60S *in the first position. *EFA*, *MAN*, and *PRO *showed BestKeeper SD values higher than 1 (Additional file 3). Altogether, the three statistical analyses pointed *ACT1 *and *60S *as the best reference genes for *A. stenosperma *qRT-PCR normalization (Table 2). These results are in accordance to our previous work with *A. stenosperma *roots using macroarray analysis [8] in which actin and *60S *were also successfully used as reference genes. *GAPDH *and β-tubulin, which previously also showed no significant variation on their expression, are here ranked in the third (M = 0.106) and fourth (M = 0.125) position, respectively, by NormFinder analysis (Additional file 3).

For *A. hypogaea*, GeNorm program indicated *ACT1 *and *UBI1 *as the most stable candidate genes (M = 0.535), whereas *PRO*, *EFA*, and *MAN *did not reach high expression stability (M > 1.5) (Table 2; Additional files 2 and 3). The pairwise variation V7/8 value (0.146) suggested the use of seven genes for normalization (Figure 2). *ACT1 *occupies the second position of the BestKeeper ranking (SD = 0.724), and *UBI2*, the first position (SD = 0.661) (Additional file 3). As for GeNorm, BestKeeper analysis considers that *PRO*, *EFA*, and *MAN *showed unstable expression (SD values higher than 1), as well as *ACT2*, *GAPDH *and *TUB*. NormFinder, differently from the other programs, ranked *60S *as the best reference gene (M = 0.045), *UBI2 *and *UBI1 *in the fifth (M = 0.107) and sixth (M = 0.121) positions, respectively, and *ACT1 *only in the eighth position (M = 0.197) (Additional file 3). In agreement with this result, Brand and Hovav [30] also considered *60S*, combined with *ADH3 *and *YLS8*, as collectively the most stable reference genes for qRT-PCR on five different *A. hypogaea *tissues, using the GeNorm and NormFinder programs. Moreover, previous studies have successfully used ubiquitin as internal reference gene for normalization of real-time data [10,11], and the elongation factor as reference gene for normalizing the transcript profiles of genes expressed following root-knot nematode exposure in *A. hypogaea *[12].

No consensus between programs was obtained for *A. duranensis*. *ACT1*/*UBI2 *(M = 0.350), *60S *(M = 0.057), and *EFA *(SD = 0.677) were indicated as the best reference genes by GeNorm, NormFinder, and BestKeeper, respectively (Table 2). However, analyzing all results together, *60S *was the best ranked gene (Additional file 3). The pairwise variation V2/3 value (0.116), calculated by GeNorm, suggested the use of *ACT1 *and *UBI2 *for normalization (Figure 2 and Additional file 3). *MAN *showed GeNorm M values higher than 1.5 indicating its unstable expression (Additional files 2 and 3). Only *EFA *and *60S *are considered as stable by BestKeeper since it presented SD values lower than 1.

A consensus was not possible for *A. magna *either. *UBI2*/*60S *(M = 0.242), *ACT2*/*PRO *(M = 0.013), and *UBI1 *(0.623) were highlighted as the most stable genes by GeNorm, NormFinder, and BestKeeper, respectively (Table 2). Considering the classification generated by the three programs, *UBI2 *followed by *60S *were the best ranked genes. The GeNorm pairwise variation V2/3 value (0.110) indicated the use of the two top ranked genes (*UBI2 *and *60S*) for normalization. *ACT2*, *TUB*, *PRO*, *EFA*, and *MAN *showed SD values, calculated by BestKeeper, higher than 1 (Figure 2 and Additional file 3) and were therefore considered unstable.

Taking into account all the dataset of the four *Arachis *species analyzed by the three programs and considering "species" as experimental subsets, we could consider that *ACT1*, *60S*, *UBI1 *and *UBI2 *were the top four reference genes and would seem very suitable as universal inter-species *Arachis *reference genes in qRT-PCR assays (Table 2; Additional file 3). There are very few reports on the selection of reference genes for gene expression studies in plant inter-species groups. However, stable references genes were established for three species of *Saccharum *spp. across different tissues [25] and a recent study indentified *GAPDH*, tubulin and 18S as the most stable reference genes for virus-infected plants of the three important cereals (wheat, barley and oats) [23]. As also observed here, these studies showed that different statistical tools not always generate the same individual gene stability values; however, the final choice of the best reference genes was almost uniform. Gutierrez and co-works [19] analyzed the stability of commonly used plant reference genes in various tissues of two models plants (*Arabidopsis thaliana *and aspen) and concluded that no gene can act as a universal reference. It was suggested a systematic validation of reference genes and the use of at least two validated reference genes involved in distinct cellular functions.

### Organ subsets

When the data was analyzed by organ subsets, roots and leaves, GeNorm and NormFinder programs pointed *ACT1 *as the most stable gene in leaves (M = 0.483 and 0.090, respectively) (Table 2). GeNorm ranked *ACT1 *and *UBI1 *as the best reference genes for leaves and generated a pairwise variation V4/5 value of 0.141 (Figure 2; Additional file 3). Only *MAN *showed GeNorm M values higher than 1.5. GeNorm and NormFinder ranks were similar, with *ACT1*, *UBI1*, and *60S *in the three first positions. BestKeeper program showed *UBI2 *as the most stable gene (SD = 0.603) (Additional file 3). However, *UBI1 *(SD = 0.807) and *ACT1 *(SD = 0.897) appeared in the second and third positions, respectively. *EFA*, *ACT2*, *GAPDH*, *PRO*, and *MAN *showed SD values higher than 1 by BestKeeper analysis.

For roots, *UBI2*/*60S *(M = 0.492), *GAPDH *(M = 0.063), and *ACT1 *(SD = 0.524) were indicated as the most stable genes by GeNorm, NormFinder, and BestKeeper, respectively (Table 2). Combining these results, *UBI2 *and *60S *were the best ranked genes, as they were also classified as good reference genes by GeNorm (first and second positions); NormFinder (fourth and sixth positions) and BestKeeper (second and third positions) (Additional file 3). GeNorm pairwise variation V3/4 value (0.126) indicated the use of the three best ranked genes (*UBI2*, *60S*, and *UBI1*) for normalization (Figure 2). All ten genes had a GeNorm M value below 1.5. *GAPDH*, *TUB*, *PRO*, and *MAN *showed SD values higher than 1, as calculated by BestKeeper (Additional file 3). In similar approaches, selection of best reference genes among samples from different tissues or organs in different plant species have enabled more accurate and reliable normalization of qRT-PCR results for gene expression studies [20,21,24]. Interestingly, *60S *and ubiquitin genes, the latter considered here as the most stable gene for both root and leaf subsets, showed quite a low level of stability in a set of five diverse peanut tissues (including roots and leaves) analyzed by GeNorm and NormFinder [30].

### Stress subsets

Analyzing the data by stress type, subsets biotic and abiotic, GeNorm and BestKeeper highlighted *ACT1 *(M = 0.549 and SD = 0.945, respectively) as the most stable gene in the samples subjected to biotic stress (Table 2). The calculated pairwise variation V3/4 value (0.145) indicated the use of the three top GeNorm ranked genes (*ACT1*, *60S*, and *UBI1*) for qRT-PCR normalization (Figure 2; Additional file 3). Only *MAN *showed an M value higher than 1.5. GeNorm and BestKeeper had very similar outcomes, pointing the same four best reference genes (*ACT1*, *60S*, *UBI1*, and *UBI2*), with a slight difference in the ranking (Additional file 3). Only *ACT1 *and *UBI2 *presented SD values lower than 1, as calculated by BestKeeper. The results generated by NormFinder program were in disagreement with those obtained by GeNorm and BestKeeper programs. NormFinder highlighted *GAPDH *as the most stable gene (M = 0.076), whilst it was ranked in the fifth (M = 0.709) and eighth (SD = 1.560) positions by GeNorm and BestKeeper, respectively. *ACT1 *appeared only in the fifth position of NormFinder classification (M = 0.130). Previous work successfully used *UBI2 *gene as a normalizer in qRT-PCR analysis of resistant *A. hypogaea *genotypes challenged to *C. personatum *[10]. In the present work, a biotic stress subset was comprised of a set of plant samples inoculated, and their respective non-inoculated controls, with pathogens that cause important diseases and reduce dramatically peanut yields. The leaves of the resistant wild peanut species *A. stenosperma *were challenged with the foliar fungus *C. personatum *and the roots with the root-knot nematode *M. arenaria *separately. The results presented here will be used in the forthcoming expression profile studies by qRT-PCR of *Arachis *candidate genes involved in these host-pathogen interactions. The further characterization of these resistance candidate genes are important steps to understand the molecular mechanisms associated with the resistance and susceptibility of wild and cultivated species of peanut, and other legumes, to fungi and nematode challenge and the introgression of resistance genes from *A. stenosperma *into the peanut crop [2,8,10,12,41].

Contrastingly, no consensus among programs was obtained for the subset abiotic stress. *UBI2*/*60S *(M = 0.376), *GAPDH *(M = 0.091), and *UBI1 *(SD = 0.464) were the most stable genes by GeNorm, NormFinder, and BestKeeper programs, respectively (Table 2). Among the three programs, *UBI2 *was the best ranked gene, appearing in the first (M = 0.376), second (M = 0.114), and third (SD = 0.682) positions by GeNorm, NormFinder, and BestKeeper, respectively (Additional file 3). GeNorm pairwise variation V2/3 value (0.136) indicated the use of *UBI2 *and *60S *for normalization (Figure 2) and only *MAN *showed M value higher than 1.5 (Additional file 3). *ACT2*, *EFA*, *TUB*, *MAN*, and *PRO *had a BestKeeper SD value higher than 1 and therefore considered as unstable genes. As for biotic stress subset, the selection of reference genes in the abiotic subset is essential for expression studies, such as characterization of *Arachis *species under drought stress, one of the most limiting factors in peanut productivity. Given the complexity of the drought response, studies of expression of genes responsive to water deficit have the potential to aid the understanding of drought tolerance mechanisms in plants [9,42].

### Reference gene validation

**Figure 3:**
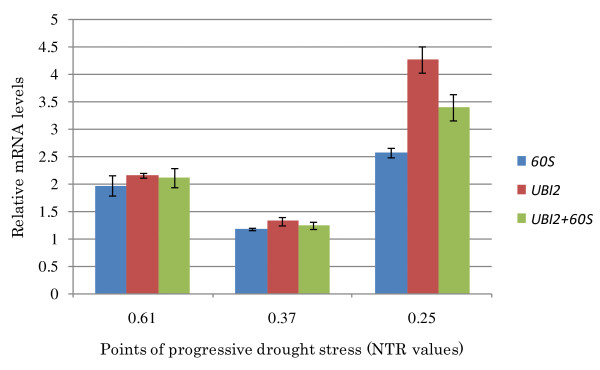
**Relative mRNA levels produced by AmDry-1 gene**. Relative mRNA levels produced by a drought inducible AmDry-1 gene in *A. magna *roots at three different stages of progressive water deficit (NTR 0.61; 0.37; and 0.25). Normalization was performed using the two most stably expressed genes, *UBI2 *and *60S*, separately or together (*UBI2*+*60S*).

To ratify the expression stability of the candidate reference genes, the expression profile of a gene induced by water deficit was analyzed using two reference genes selected in this study. The target gene (AmDry-1) was selected from a subtractive cDNA library of *A. magna *roots submitted to a gradual water deficit in soil and showed to be overexpressed *in silico *and by RT-PCR analysis in drought conditions (unpublished data). The expression level of AmDry-1 was assessed in *A. magna *roots at three distinct stages of progressive water deficit treatment based on the estimate NTRs (0.61; 0.37 and 0.25, respectively), using *60S *and *UBI2 *as reference genes, as they were the two most stably expressed in this species, in roots and in abiotic stress treatment (Table 2). A comparison between Cq values of stressed and control plants from all analyzed stages of stress was conducted for *UBI2 *and *60S *data that showed a normal pattern of distribution when evaluated by Shapiro-Wilk tests (W = 0.927, P = 0.347 for *UBI2 *and W = 0.907, P = 0.196 for *60S*). ANOVA analysis showed that Cq values of both reference genes did not differ significantly between stressed and control plants (F = 0.002, P = 0.963 and F = 2.766, P = 0.127; for *UBI2 *and *60S*, respectively), confirming the stable expression of these genes between treatments (stressed and control) and different stages of stress. Similar expression patterns of the target gene were obtained when *UBI2 *or *60S *was used for normalization. Nevertheless, estimated transcript abundance was higher when values were normalized against *UBI2 *than with *60S *(Figure 3). When both genes were used together for normalization, intermediate values were obtained and the differences in transcript abundance between the two reference genes might explain these results [26]. Target gene expression was also analyzed statistically and the normalized Cq values, ΔCq (Cq target gene - Cq reference gene) of control and stressed plants were compared by using Kruskal-Wallis tests, a non parametric test, as ΔCq data did not show a normal pattern of distribution. Analyses were made with target genes Cq values normalized with *UBI2 *and *60S *reference genes. The results showed that ΔCq differ significantly between stressed and control plants (chi-square = 6.564, df = 1.000, P = 0.010 for *UBI2 *and chi-squared = 3.692, df = 1.000, P = 0.055 for *60S*), confirming the previously detected overexpression of the target gene (AmDry-1) during plant response to drought treatment.

## Conclusions

We have assessed the stability of ten candidate reference genes for qRT-PCR normalization using an entire dataset and eight samples subsets of leaves and roots from wild relatives and cultivated peanut species submitted to biotic and abiotic stresses. For that, we used the three most commonly used statistical programs, GeNorm, NormFinder, and BestKeeper. It is the first in-depth study of reference genes validation in wild *Arachis *species and will allow the use of specific combinations of reference genes for the quantification of mRNA by qRT-PCR in complex experimental conditions. In each of the eight sample subsets studied here, a combination of two reference genes involved in different cellular processes was identified as a suitable standard. The use of the reference genes characterized here should improve the accuracy and reliability of gene expression analysis across various organs and type of stresses in different *Arachis *species, contributing particularly for the understanding of stress tolerance/resistance mechanisms in legumes.

## Competing interests

The authors declare that they have no competing interests.

## Authors' contributions

CVM carried out the qRT-PCR assays, performed the statistical analysis and drafted the manuscript; PMG participated in conceiving the study, data analysis and drafting the manuscript; ACQM conducted greenhouse assays and data analysis; ACGA conducted greenhouse assays and data analysis; SCMLB conducted greenhouse assays and data analysis; DJB participated in conceiving the study and drafting the manuscript; ACMB conceived of the study, and participated in its design and coordination and drafted the manuscript. All authors read and approved the final manuscript.

## Supplementary Material

Additional file 1 **Dissociation curve of the ten reference genes**. Dissociation curve generated for each reference gene tested: (A) *UBI1*; (B) *ACT1*, (C) *ACT2*; (D) *UBI1*; (E) *TUB*; (F) *MAN*; (G) *GAPDH*; (H) *EFA*; (I) *PRO*; (J) *60S*. X-axis: Temperature (°C); Y-axis: Derivative reporter (-Rn). Click here for file

Additional file 2 **Expression stability for the ten reference genes analyzed by the GeNorm software**. Analysis on the (A) entire dataset and individual subsets: (B) *A. stenosperma*; (C) *A. duranensis*; (D) *A. magna*; (E) *A. hypogaea*; (F) leaves; (G) roots; (H) biotic stress; (I) abiotic stress. Average expression stability values M (Y-axis) of the candidate reference genes are plotted from the least stable to the most stable (X-axis). Click here for file

Additional file 3 **Ranking of candidate genes based on their expression stability values estimated by GeNorm, NormFinder, and BestKeeper**. Analysis conducted with the entire dataset and individual (species, organ or stress) subsets. Click here for file
