# Supplementary figures and images for: Reference genes for quantitative reverse transcription-polymerase chain reaction expression studies in wild and cultivated peanut
Source: BMC Res Notes. 2011 Sep 9;4:339. doi: 10.1186/1756-0500-4-339 (PMC3180468; doi:10.1186/1756-0500-4-339)

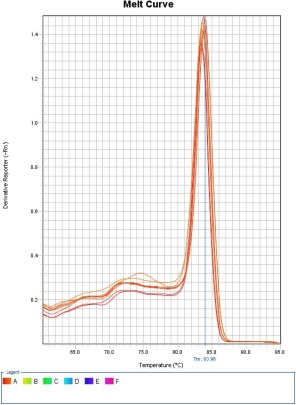

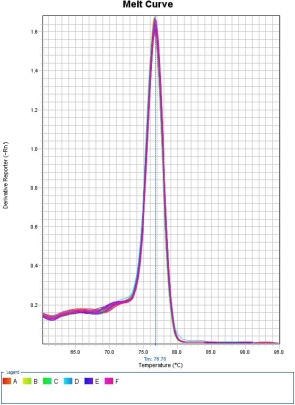

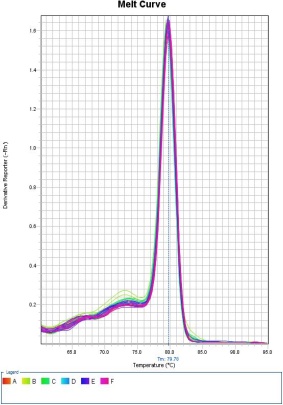

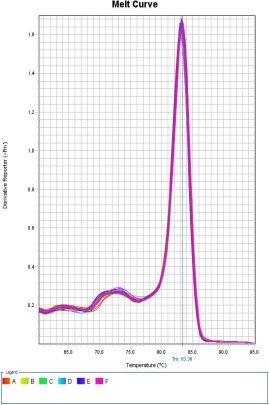

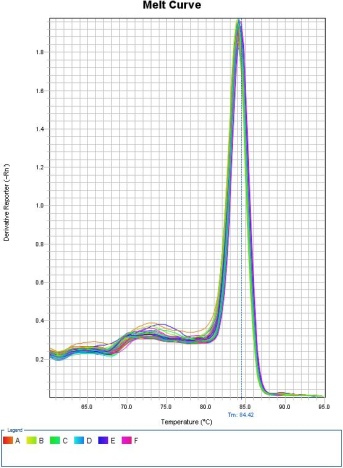


A

B

C

D


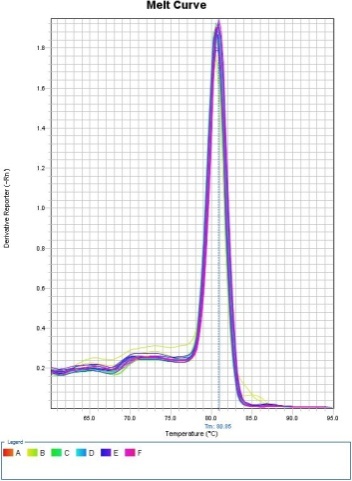

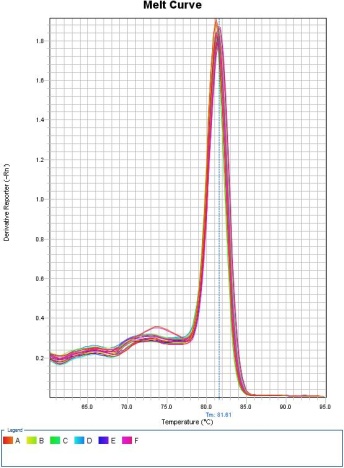


E

F

G

H


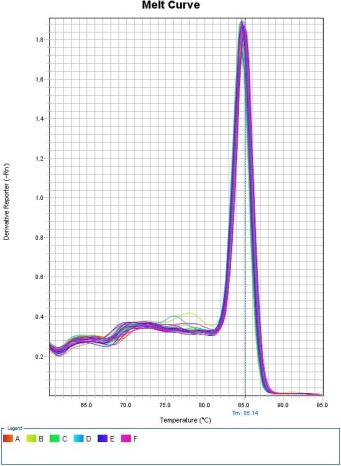


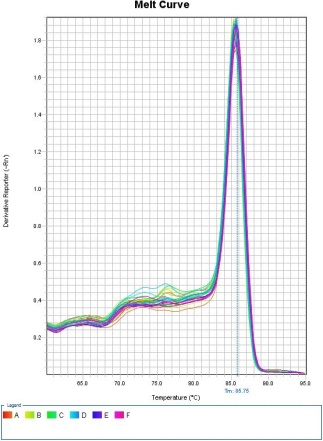

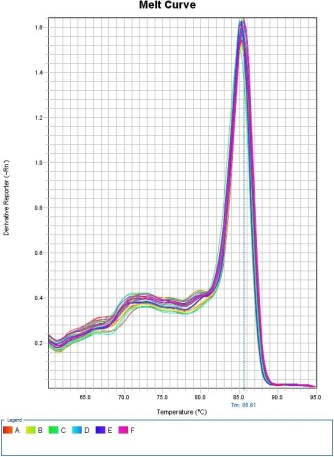


I

J

Supplement: Additional file 1 — Dissociation curve of the ten reference genes. Dissociation curve generated for each reference gene tested: (A) UBI1; (B) ACT1, (C) ACT2; (D) UBI1; (E) TUB; (F) MAN; (G) GAPDH; (H) EFA; (I) PRO; (J) 60S. X-axis: Temperature (°C); Y-axis: Derivative reporter (-Rn). [file 1756-0500-4-339-S1.DOC]

## Slide 1
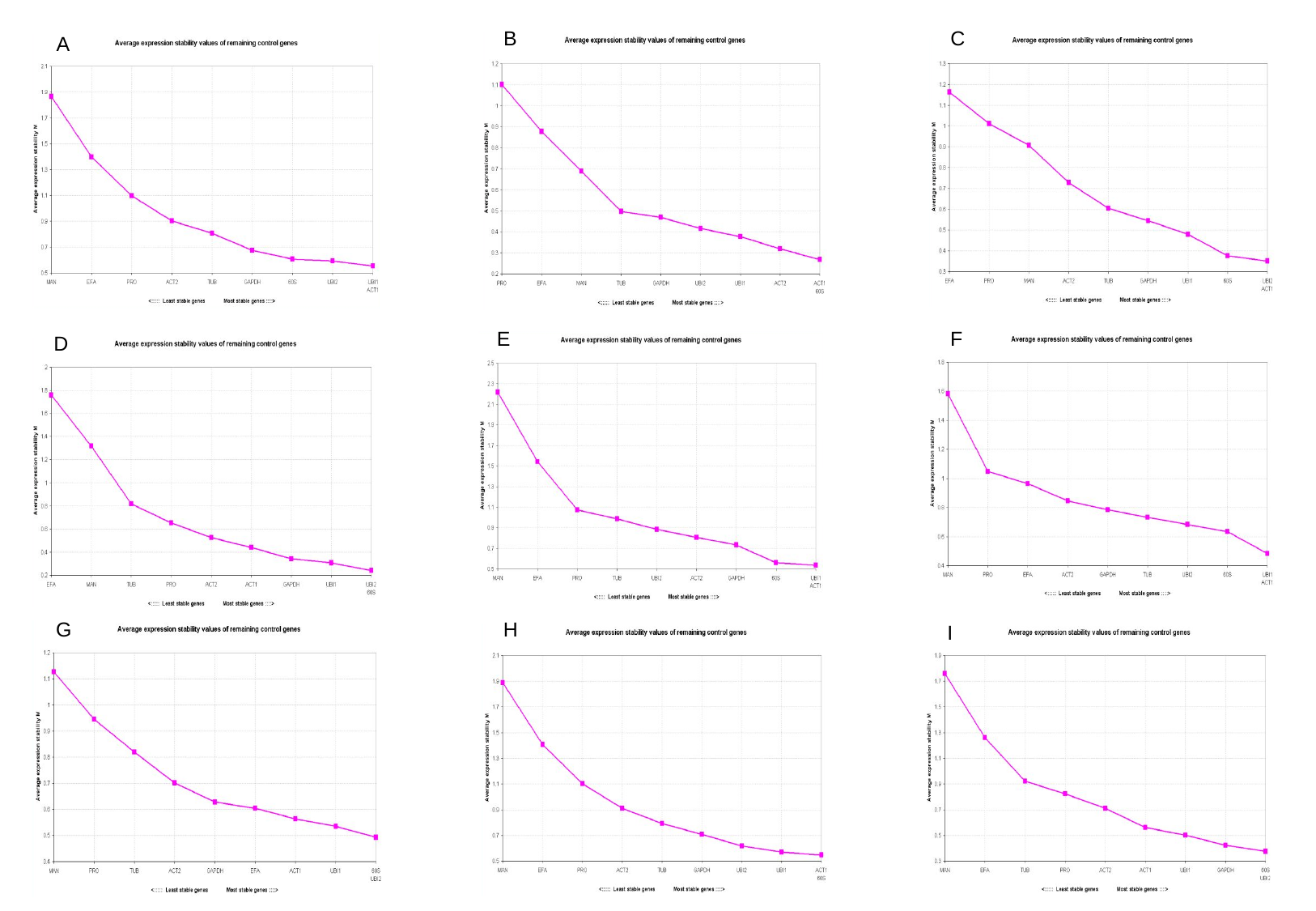

B
C
A
E
F
D
G
H
I

Supplement: Additional file 2 — Expression stability for the ten reference genes analyzed by the GeNorm software. Analysis on the (A) entire dataset and individual subsets: (B) A. stenosperma; (C) A. duranensis; (D) A. magna; (E) A. hypogaea; (F) leaves; (G) roots; (H) biotic stress; (I) abiotic stress. Average expression stability values M (Y-axis) of the candidate reference genes are plotted from the least stable to the most stable (X-axis). [file 1756-0500-4-339-S2.PPT]
